# Mother-to-infant transmission of the carcinogenic colibactin-producing bacteria

**DOI:** 10.1186/s12866-021-02292-1

**Published:** 2021-08-24

**Authors:** Yuta Tsunematsu, Koji Hosomi, Jun Kunisawa, Michio Sato, Noriko Shibuya, Emiko Saito, Haruka Murakami, Yuko Yoshikawa, Yuji Iwashita, Noriyuki Miyoshi, Michihiro Mutoh, Hideki Ishikawa, Haruhiko Sugimura, Motohiko Miyachi, Keiji Wakabayashi, Kenji Watanabe

**Affiliations:** 1 Department of Pharmaceutical Sciences, University of Shizuoka, 422-8526 Shizuoka, Japan; 2 Laboratory of Vaccine Materials, Center for Vaccine and Adjuvant Research, Laboratory of Gut Environmental System, Health and Nutrition (NIBIOHN), National Institutes of Biomedical Innovation, 567-0085 Ibaraki-city, Japan; 3 Department of Pediatrics, Maternal and Child Health Center, Aiiku Clinic, 106-8580 Tokyo, Japan; 4 Department of Human Nutrition, Tokyo Kasei Gakuin University, 194-0292 Tokyo, Japan; 5 Department of Physical Activity Research, Health and Nutrition (NIBIOHN), National Institutes of Biomedical Innovation, 162-8636 Tokyo, Japan; 6 School of Veterinary Medicine, Faculty of Veterinary Science, Nippon Veterinary and Life Science University, 180-8602 Tokyo, Japan; 7 Department of Tumor Pathology, Hamamatsu University School of Medicine, 431- 3192 Shizuoka, Japan; 8 Graduate School of Nutritional and Environmental Sciences, University of Shizuoka, 422-8526 Shizuoka, Japan; 9 Department of Molecular-Targeting Cancer Prevention, Kyoto Prefectural University of Medicine, 602-8566 Kyoto, Japan

**Keywords:** Colibactin, Natural product, *Escherichia coli*, Perinatal transmission, Colorectal cancer

## Abstract

**Background:**

The *Escherichia coli* strain that is known to produce the genotoxic secondary metabolite colibactin is linked to colorectal oncogenesis. Therefore, understanding the properties of such colibactin-positive *E. coli* and the molecular mechanism of oncogenesis by colibactin may provide us with opportunities for early diagnosis or prevention of colorectal oncogenesis. While there have been major advances in the characterization of colibactin-positive *E. coli* and the toxin it produces, the infection route of the *clb* + strain remains poorly characterized.

**Results:**

We examined infants and their treatments during and post-birth periods to examine potential transmission of colibactin-positive *E. coli* to infants. Here, analysis of fecal samples of infants over the first month of birth for the presence of a colibactin biosynthetic gene revealed that the bacterium may be transmitted from mother to infant through intimate contacts, such as natural childbirth and breastfeeding, but not through food intake.

**Conclusions:**

Our finding suggests that transmission of colibactin-positive *E. coli* appears to be occurring at the very early stage of life of the newborn and hints at the possibility of developing early preventive measures against colorectal cancer.

**Supplementary Information:**

The online version contains supplementary material available at 10.1186/s12866-021-02292-1.

## Background

Colorectal cancer (CRC) is the third most common form of cancer and the second most common cause of cancer mortality in the world [1]. It is predicted that by 2030 approximately 2.2 and 1.1 million people will develop CRC and succumb to it [2]. To reduce the number of CRC incidences and mortalities, it is vital to identify and mitigate the source of risk factors that contribute to the onset of CRC. Certain strains of *Escherichia coli* that harbor the gene cluster *clb* (also referred to as *pks*) responsible for the biosynthesis of the genotoxin colibactin have been linked to colorectal oncogenesis [3–10]. Recently, we created fluorescent probes [11] that are turned on specifically by ClbP, a peptidase required to activate the prodrug-like colibactin precursor [12, 13]. The probe allowed high-throughput screening of *E. coli* isolates from clinical samples, which led to the isolation of the high-colibactin producer *E. col*i-50 that will be useful in studying the properties of colibactin-positive (*clb*+) *E. coli* and colibactin [11]. While we continue to elucidate the molecular mechanism of oncogenesis by *clb* + *E. coli* and colibactin, there is still a limited understanding on the infection route of the *E. coli* to the affected individuals. Since identification of the routes of infection would help develop measures to mitigate or prevent the infection, we initiated a screening effort to examine the prevalence of *clb* + *E. coli* among healthy individuals by analyzing their fecal samples [14] and are currently investigating the continued *clb* + *E. coli* infections that occur among healthy individuals. Also, it is known that the newborn gut microbiota starts to form upon exposure to the vaginal and maternal skin microbiomes after birth [15]. A study based on a rat model showed that commensal *E. coli* strains, including clb + *E. coli*, are transmitted from mothers to neonates, where early colonization of neonate gut with genotoxic *E. coli* could influence the intestinal homeostasis at adulthood in a way that may put the individual at risk of colorectal cancer and other immune-mediated diseases [16]. Therefore, we extended our screening to include newborns to study our main clinical objective, which is to determine if *clb* + *E. coli* could be transmitted to infants from mother or closely interacting caretaking adults, and if so, what is the source and the medium through which the strain is being passed on to the infants. Here we report that the colibactin-producing *E. coli* can indeed be rapidly transmitted from mother to child after birth, suggesting that a respectable number of healthy individuals may become predisposed to high risk of CRC at the very early stage of life.

## Results

**Fig. 1:**
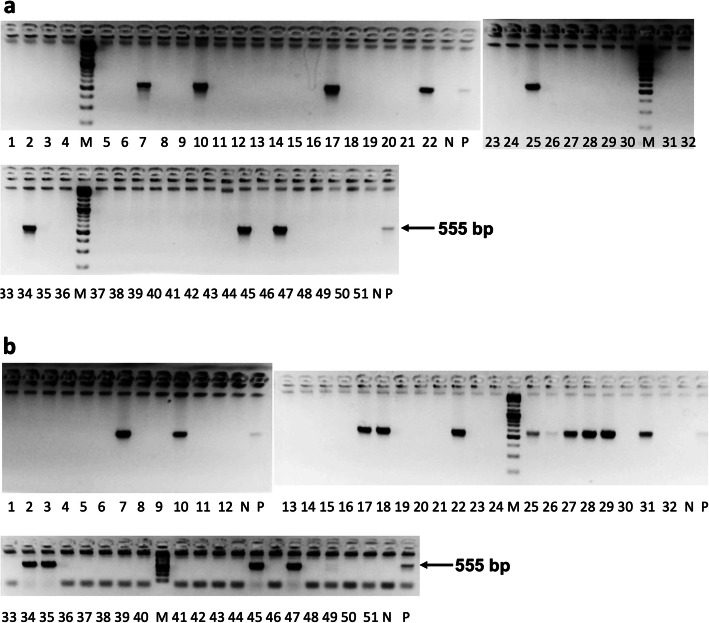
PCR analysis for the presence of *clb* + *E. coli* in fecal samples of infants. Amplifications of PCR products from the DNA samples extracted from fecal samples was performed using the primer set described in Online Methods. (**a**) Results from the fecal samples from infants collected at birth to two to three days after birth. (**b**) Results from the fecal samples collected one month after birth. Lane M: molecular weight marker; lane N: negative control using pUC19 as a PCR template; lane P: positive control using the gDNA isolated from *E. coli*-50[11] as a PCR template; lanes 1–51: analysis of the sample from each of the 51 newborn subjects. The size of the expected *clbB* amplicon generated with the primer set clb-F/clb-R is 555 bp. Full-length gels are presented in Supplementary Fig. 1 of the Supplementary Information

**Fig. 2:**
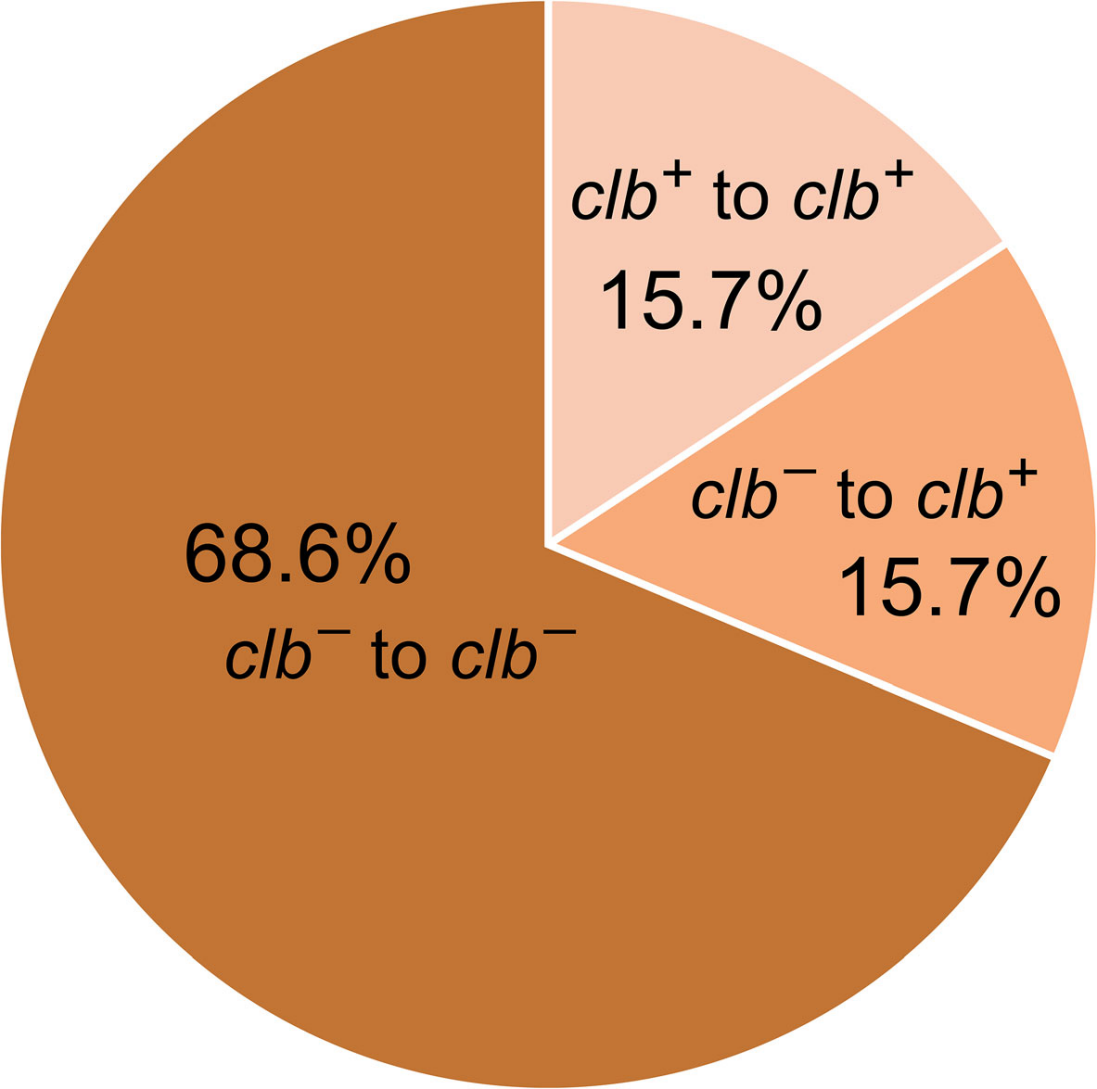
The frequency of diagnosis for *clb* + and *clb*– among the newborns. The *clb*-positive rate among the newborns at birth to a few days after birth is 15.7 %, and one month that is 15.7 % (*clb* + to *clb*+) plus 15.7 % (*clb–* to *clb*+), 31.4 % in total. Infants were screened within two to three days of birth after one month of either mixed feeding or breastfeeding alone

In a previous study we conducted to identify the frequency of healthy adults who carry *clb* + *E. coli*, we surveyed 223 healthy adults from Tokyo metropolitan area in Japan for the presence of *clb* + *E. coli* in their fecal samples. We found that 60 participants (26.9 %) were positive [14]. We extended the study further to investigate the timing at which individuals become infected with *clb* + *E. coli*. For the current study, we examined 51 infants (25 male, 26 female). From the subjects, one set of feces was collected at birth or within two to three days after birth, and another set was collected one month after birth. The samples were examined by PCR to detect the presence of *clbB*, the gene for one of the PKS–NRPS hybrid megasynthetases encoded in the colibactin biosynthetic gene cluster. We found that 8 out of 51 newborns or 15.7 % of the test samples harbored *clb* + *E. coli* immediately after birth (Fig. 1a, lanes 7, 10, 17, 22, 25, 34, 45 and 47 and Fig. 2). On the other hand, 16 of the 51 (31.4 %) tested positive for *clb* + *E. coli* one month after birth (Fig. 1b, lanes 7, 10, 17, 18, 22, 25, 26, 27, 28, 29, 31, 34, 35, 45, 47 and 49 and Fig. 2), indicating that 8 newborns or 15.7 % acquired the *clb* + *E. coli* strain during their first month. The *clb*-positive rate increased from 15.7 to 31.4 % by the end of the first month, reaching to the equivalent level of 26.9 % observed among healthy adults examined recently [14].

Perinatal transmission can happen *in utero*, in the birth canal or through breastfeeding. Because intrauterine transmission of *E. coli* in healthy pregnancies is considered to be infrequent [17], we next investigated the correlation between the method of delivery and the *clb*-positive ratio among the newborns we studied. Because we expected that the delivery method would only affect the *clb*-positive ratio among newborns to a few-days-old babies, we did not employ the data collected one month after birth. Regarding the birth canal transmission, *clb* + *E. coli* was detected in seven out of eight or 87.5 % of the infants that were born through natural delivery (Table 1). In contrast, only one of eight or 12.5 % of the infants that were delivered by Cesarean section was *clb*-positive (Table 1).

**Table 1:** The correlation between the frequency of infection with *clb* + *E. coli* among 51 infants examined and their delivery methods. Number of infants (*n* = 51)

	No. of infants (*n* = 51)				
Delivery method	*clb*+	*clb–*	Total	OR (95 % CI)	*p*-value
Natural delivery	7	35	42	1.6 (0.17 to 14.90)	0.67
Cesarean section	1	8	9	1	

OR: odds ratio, CI: confidence interval

As to the breastfeeding-mediated transmission, we examined the correlation between the infant feeding mode and the *clb*-positive ratio. We screened the 43 infants who were determined to be *clb*-negative at birth to a few days after birth. After one month, eight of the 43 *clb*-negative infants presented *clb*-positive, while the remaining 35 (81.4 %) were not affected as determined by the PCR analysis of their fecal samples (Table 2). In total, there were 26 and 17 infants who were fed breastmilk alone and a mixture of formula and breastmilk, respectively. Among the eight *clb*-positive infants, seven were breastfed strictly over the one-month period, whereas only one newborn was fed a mixture of formula and breastmilk over the month. While 26.9 % of those given breastmilk alone became *clb*-positive, only 5.9 % of the infants given a mixed feed became *clb*-positive (Table 2). To check if intaking or handling of food items by caretakers could play a role in transmitting *clb* + *E. coli* to the infants, 58 different samples collected from food items including tap water that are commonly consumed by the demographics examined in the current study were screened for the presence of *clb* + *E. coli* (Table 3). The search failed to identify *clb* + *E. coli* except in a sample of cattle stomach. However, the strain found in the sample of cattle stomach belonged to the phylogroup B1, whereas all of the *clb* + *E. coli* strains isolated from human subjects thus far belonged to B2 (manuscript in preparation, Y.Y., Y.T., M.S., Y.I., N.M., M.Mutoh., H.I., H.S., K.Wakabayashi and K.Watanabe). Lastly, we did not observe any difference in the *clb* + *E. coli* infection ratio between the sexes of the infants either at birth or one month after birth in this study.

**Table 2:** The correlation among the frequency of diagnosis for *clb* + vs. *clb*– and provision of one month of mixed feeding vs. breastfeeding alone among the newborns who tested negative at birth or two to three days after birth. For each category, breakdown of the cases based on delivery methods is also given. Number of infants (*n* = 43)

	No. of infants (*n* = 51)				
Feeding	*clb*+	*clb–*	Total	OR (95 % CI)	*p*-value
Breastfeeding alone	7	19	26	5.89 (0.65 to 53.11)	0.11
Natural delivery	7	16	23		
Cesarean section	0	3	3		
Mixed feeding with breastmilk and formula	1	16	17	1	
Natural delivery	1	11	12		
Cesarean section	0	5	5		

OR: odds ratio, CI: confidence interval

**Table 3:** Screening of 58 food samples commonly consumed in Japan for *clb* + *E. coli* by PCR analysis

**Source**	**PCR**	**Source**	**PCR**	**Source**	**PCR**
**Vegetable**					
bran pickles	**–**	broccoli	**–**	brown beech mushroom	**–**
burdock root	**–**	cabbage	**–**	celery	**–**
cherry tomato	**–**	Chinese cabbage	**–**	cloud ear mushroom	**–**
cucumber	**–**	dried shiitake mushroom	**–**	enoki mushroom	**–**
eryngii mushroom	**–**	ginger	**–**	Japanese ginger	**–**
Japanese parsley	**–**	Japanese radish	**–**	kimchi	**–**
Korean lettuce	**–**	lettuce	**–**	lotus root	**–**
maitake mushroom	**–**	mesclun greens	**–**	olive oil	**–**
onion	**–**	parsley	**–**	pea sprouts	**–**
pickled Chinese cabbage	**–**	potherb mustard	**–**	red leaf lettuce	**–**
salted plum	**–**	shiitake mushroom	**–**	soybean sprouts	**–**
spinach	**–**	spring onion	**–**	tomato	**–**
white radish sprouts	**–**				
**Meat**					
beef	**–**	beef / ground meat	**–**	cattle / large intestine	**–**
cattle / liver	**–**	cattle / small intestine	**–**	cattle / stomach	**+**
chicken / ground meat	**–**	chicken / liver	**–**	pork / ground meat	**–**
**Seafood**					
clam	**–**	crab	**–**	freshwater clam	**–**
salmon	**–**	shrimp	**–**	tuna	**–**
**Dairy products**					
butter	**–**	cheese	**–**	milk	**–**
**Others**					
honey	**–**	tap water	**–**	yogurt	**–**

## Discussion

Our survey of infants identified that only 15.5 % of the screened infants harbored *clb* + *E. coli* immediately after birth (Fig. 1a, lanes 7, 10, 17, 22, 25, 34, 45 and 47 and Fig. 2). However, after one month the percentage doubled to 31.4 %, similar to the frequency found among healthy adults [14] (Fig. 1b, lanes 7, 10, 17, 18, 22, 25, 26, 27, 28, 29, 31, 34, 35, 45, 47 and 49 and Fig. 2). Once infected, it is expected that *clb* + *E. coli* remains within the system of the infected individual persistently. Thus, the adult-like *clb*-positive rate we found among the one-month-old newborns in this study suggests that healthy individuals become infected with *clb* + *E. coli* very early in their life stage in Japan. These results also indicate that the infants are getting exposed to the source of *clb* + *E. coli* under the ordinary living condition during its first month of life.

Next, we examined the source of perinatal transmission, where we focused on the correlation between the method of delivery and the *clb*-positive ratio among the newborns. The study found that while 87.5 % of the infants delivered by natural birth were *clb*-positive, only 12.5 % of those delivered by Cesarean section were *clb*-positive (Table 1). Those results indicated that a higher *clb*-positive ratio was observed among infants that were born through natural childbirth, similar to how the chance of infants acquiring the vaginal flora bacteria, including *E. coli*, increases by passing through the birth canal.

We also examined the possible role breastfeeding plays in the transmission of *clb*-positive *E. coli* to infants. Of the *clb*-negative infants that were strictly breastfed, 26.9 % became *clb*-positive one month after birth, while only 5.9 % of those given a mixture of formula and breastmilk turned *clb*-positive (Table 2). A simple survey of food items that are considered to be consumed typically by the demographics to which infant caretakers belong showed that none of the food samples examined was contaminated by the *clb* + *E. coli* strains that are isolated from human subjects. Therefore, intaking or handling of food items by caretakers being a potential source of *clb* + *E. coli* being transmitted to infants was considered less likely. The results implicate that the transmission of *clb*-positive *E. coli* to infants occurs mainly through mothers having close contacts with the infants.

## Conclusions

In summary, the infection ratio of *clb* + *E. coli* is 31.4 % among the one-month-old infants studied, which is similar to the frequency found among healthy adults [14]. It was also reported that 130 Swedish infants followed from birth to 18 months of age were determined to be 33 % *clb*-positive, very similar to our findings of 31.4 %, when their feces were analyzed using a similar PCR method [18]. Our analysis identified that infants born by natural delivery had a higher incidence of being *clb*-positive than those born by Cesarean section. Similarly, those who were breastfed strictly showed a higher *clb*-positive frequency than those given a mixed feed. The fact that the mixed feed also contained breastmilk suggests that breastmilk itself was not the source of *clb* + *E. coli*. A simple survey of food items commonly consumed by the Japanese also indicated that *clb* + *E. coli* was probably not transmitted through contaminated food consumed or handled by caretakers of the infants. Rather, the most probable route of infection of the potentially oncogenic *clb* + *E. coli* strain appears to be through direct skin-to-skin contact, or skin-to-mouth contact involved in breastfeeding to be more specific, between mother and her infant (Table 2). The same can be said about the method of delivery, where infants born by natural birth would have a substantially higher frequency of direct skin-to-skin contact with its mother than those born by Cesarean section (Table 1). Taken together, similar to how the gut microbiota is transmitted from mother to infant [19], our results strongly imply that *clb* + *E. coli* might be transmitted from mother to newborn from the very early stage of life of the newborn through intimate contacts with the mother. Therefore, by implementing measures that can reduce the transmission of *clb* + *E. coli* from adults to infants, we may be able to lower the incidence of CRC in our population. For instance, we would be able to develop early preventive measures against colorectal cancer. Those measures could include procedures to prevent infection during childbirth, such as providing counselling to expecting couples to help them become aware of the condition, testing the vaginal flora and altering the flora by external interferences [20] or providing couples assistance in choosing Cesarean section for delivering their infants. Of course, if future research establishes safe treatments to eradicate *clb* + *E. coli*, such procedures could be performed before pregnancy or at a stable time during pregnancy to reduce the mother-to-infant transmission of *clb* + *E. coli*. To this end, we are currently analyzing the fecal samples from the mothers of the infants to fully understand the rates and modes of *clb* + *E. coli* transmission from mothers and her infants.

## Methods

### Participants and sample collection

The subjects were 51 healthy infants from Tokyo metropolitan area in Japan. The size of the subject pool was deemed adequate for the study based on the known frequencies of *clb* + individuals found among healthy individuals (approximately 26.9 % [14]) and child deliveries being carried out by Cesarean Sec. (24.4 % for 2005–2015 [21], and the rate is steadily increasing [22]) in Japan. If the frequency of *clb* + infants is similar to that of healthy adults, approximately three to four *clb* + infants are expected among 51 subjects (51 ⋅ 0.269 ⋅ 0.244 = 3.3). Fecal samples were collected at birth to two to three days after birth (at the time of discharge within a few days after birth) and one month after birth (at the time of the one-month checkup). The collected feces were immediately placed in a sealed container and stored in a − 20 °C freezer until DNA extraction was performed.

### DNA extraction from fecal samples

DNA was extracted from the frozen fecal samples with the bead beating method using a GNOME DNA Isolation Kit (MP Biomedicals). DNA quality was assessed with an Agilent 4200 TapeStation (Agilent Technologies). After the final DNA precipitation step, the DNA samples were resuspended in TE buffer and stored at − 80 °C before the PCR analysis.

### DNA extraction from food materials

Each food sample (10–50 g) obtained from grocery stores in Shizuoka, Japan, was added to 20 mL of EC medium (20 g peptone, 5 g lactose, 1.5 g bile salt, 4 g K_2_HPO_4_, 1.5 g KH_2_PO_4_ in 1 L H_2_O) in a sterilized bag. The mixture was incubated at 44.5 °C for 24 h. The mixture was filtered, and the filtrate was centrifugated at 10,000 *g* for 6 min at 4 °C. The collected precipitate was suspended in 1 mL of sterile Milli-Q water. The suspension was plated on a MacConkey agar medium and incubated at 37 °C for 16 h. The grown bacteria were cultured, and its genomic DNA was extracted by using a DNA isolation kit (QIAGEN). The isolated DNA was resuspended in TE buffer and stored at − 80 °C before the PCR analysis.

### Confirmation of the presence of the *clb* gene cluster by PCR

The extracted fecal DNA was subjected to PCR (SapphireAmp Fast PCR Master Mix, Takara) and qualitatively analyzed for *clbB* (a 9.6-kilobase gene encoding one of the colibactin biosynthetic enzymes) present in the DNA extract by amplifying the gene fragment using a primer set of clbB-F: 5’-TGTTCCGTTTTGTGTGGTTTCAGCG-3’ and clbB-R: 5’-GTGCGCTGACCATTGAAGATTTCCG-3’ as described previously [11]. The correlation was analyzed by comparing the presence or absence of the *clbB* gene with the subject’s birth method, diet content and sex.

### Statistical analysis

Each experiment was performed at least three independent times. Representation of data as dot-plots and bar-and-whisker graphs is described in figure legends. The t test for determining the statistically difference between the expected and observed frequencies was calculated using JMP (SAS Institute Inc.) and the NORM.DIST function in Microsoft Excel version 16.16.25.

## Supplementary Information

**Additional file 1.**

## Data Availability

The datasets used and/or analyzed during the current study are available from the corresponding author on reasonable request.
