# Supplementary material for: Mother-to-infant transmission of the carcinogenic colibactin-producing bacteria
Source: BMC Microbiol. 2021 Aug 24;21:235. doi: 10.1186/s12866-021-02292-1 (PMC8386082; doi:10.1186/s12866-021-02292-1)
Supplement: Supplementary file 1 — Additional file 1. [file 12866_2021_2292_MOESM1_ESM.docx]

**Supplementary Information for**

**Mother-to-infant transmission of the carcinogenic colibactin-producing bacteria**

Yuta Tsunematsu,^1^ Koji Hosomi,^2^ Jun Kunisawa,^2^ Michio Sato,^1^ Noriko Shibuya,^3^ Emiko Saito,^4^ Haruka Murakami,^5^ Yuko Yoshikawa,^6^ Yuji Iwashita,^7^ Noriyuki Miyoshi,^8^ Michihiro Mutoh,^9, 10^ Hideki Ishikawa,^10^ Haruhiko Sugimura,^7^ Motohiko Miyachi,^5^ Keiji Wakabayashi,^8^ and Kenji Watanabe*^,1^

* Corresponding author at: Department of Pharmaceutical Sciences, University of Shizuoka, Shizuoka, 422-8526, Japan. Tel: +81-54-264-5662. Fax: +81-54-264-5666. E-mail: kenji55@u-shizuoka-ken.ac.jp

***Correspondence e-mail: kenji55@u-shizuoka-ken.ac.jp**

**Supplementary Results**

**Supplementary Figure 1.** Photographs of the DNA agarose gels without cropping shown in **Figure 1**.
